# Moderators uncertainty tolerance (UT) in healthcare: a systematic review

**DOI:** 10.1007/s10459-023-10215-0

**Published:** 2023-04-25

**Authors:** Ana Yap, Priscilla Johanesen, Chris Walsh

**Affiliations:** 1 Department of Microbiology, Biomedical Discovery Institute, Monash University, Clayton, Victoria 3800 Australia; 2 Victoria University, Victoria University (VU) Online, Melbourne, VIC Australia

**Keywords:** Uncertainty, Tolerance, Moderators, Responses, Systematic review, Healthcare professionals, Qualitative

## Abstract

Uncertainty tolerance (UT) is integral to healthcare. Providers’ responses to medical uncertainty has ramifications on the healthcare system, the healthcare provider and the patient. Understanding healthcare providers’ UT, is important for improving patient-care outcomes. Understanding whether and to what extent it is possible to modulate individuals’ perceptions and responses to medical uncertainty, can provide insights into mechanisms for support for training and education. The objectives of this review were to further characterize moderators of healthcare UT and explore moderator influences on the perceptions and responses to uncertainty experienced by healthcare professionals. Framework analysis of qualitative primary literature was conducted on 17 articles, focusing on the impacts of UT on healthcare providers. Three domains of moderators were identified and characterized relating to the healthcare provider’s personal attributes, patient-derived uncertainty and the healthcare system. These domains were further categorized into themes and subthemes. Results suggest these moderators influence perceptions and responses to healthcare uncertainty across a spectrum ranging from positive to negative to uncertain. In this way, UT could be a state-based construct within healthcare settings and is contextually determined. Our findings further characterize the integrative model of uncertainty tolerance (IMUT) (Hillen Social Science and Medicine 180, 62–75, 2017) and provide evidence for the relationship between moderators and their influences on cognitive, emotional and behavioral responses to uncertainty. These findings provide a foundation for understanding the complex nature of the UT construct, add to theory development, and provide groundwork for future research exploring appropriate support for training and education in healthcare fields.

**Supplementary Information:**

The online version contains supplementary material available at 10.1007/s10459-023-10215-0.

## Introduction

Uncertainty tolerance (UT) refers to an individual’s ability to cope with perceived ambiguity or uncertainty. UT is most often defined as a psychological construct that describes the relationship between an individuals’ perceptions, emotions and behaviors towards ambiguous events or stimuli (Budner, 1962). Uncertainty is an inherent and pervasive part of healthcare (Babrow et al., 1998; Fox, 1980; Han et al., 2011), and has negative effects on patient care and staff wellbeing (Farnan et al., 2008; Hancock & Mattick., 2020; Hillen et al., 2017; Katz, 1984). Despite research beginning in the mid-twentieth century into the nature of people’s management and responses to uncertainty (Budner, 1962; Frenkel-Brunswik, 1949), the field is only just beginning to uncover the complexities related to how individuals manage uncertainty within the healthcare setting. Consequently, improved understanding of healthcare-related UT, or how healthcare providers perceive and/or respond to uncertain and/or ambiguous stimuli within the healthcare setting, warrants further exploration (Hillen et al., 2017; Strout et al., 2018).

A healthcare providers’ inability to cope with medical uncertainty may result in excessive diagnostic testing, unnecessary admissions of patients for observation; and/or inappropriate patient referrals, all of which have implications for the healthcare system, healthcare provider and patient-care outcomes (Bachman & Freeborn, 1999; Farnan et al., 2008; Katz, 1984; Kim & Lee, 2018; Lawton et al., 2019). However, there are nuances within UT that are complex and extend beyond whether having higher UT is good or bad. For example, it is possible that a provider with higher UT could lapse into contentment of their circumstances which could result in poor practice, or they could be proactive and problem-solve issues that arise (Reis-Dennis et al., 2021). Despite the reported importance of the UT construct in healthcare practice, a framework for effectively fostering healthcare providers’ UT, remains elusive.

A challenge to advancing the field may be related, at least in part, to the ongoing debate as to whether UT is a static personality trait (Koerner & Dugas, 2008), or a dynamic and modifiable state (Durrheim & Foster, 1997; Herman et al., 2010). Hillen et al. (2017) developed a contemporary and comprehensive integrative model of uncertainty tolerance (IMUT) and suggested that exploration of UT as a trait or a state are *both* appropriate. Some literature alludes to UT being predominately a psychological trait (Koerner & Dugas, 2008), as such, these studies typically view UT as a measurable and stable construct and often omit exploring the context-specific manifestations of uncertainty (Geller et al., 1990, 1993; Hillen et al., 2017). In the literature, where UT is explored as a modifiable state, the state of UT is determined by either contextual or situational factors that may change the condition of the individuals’ UT (Durrheim & Foster, 1997; Herman et al., 2010). This signals a trait-focused approach is suitable for characterizing and understanding UT, whereas the state-focused approach is suitable for exploring contextual factors that influence UT (Hillen et al., 2017). In an effort to enhance and extend the existing IMUT, this systematic review explores the context-specific manifestations of UT by characterizing moderators that may influence an individual healthcare provider’s UT. The IMUT, in addition to providing a conceptual model for the entire pathway of the uncertainty tolerance construct from stimulus through to response, makes provisions for moderators. Moderators were initially proposed by Hillen et al. (2017) as contextual and situational factors influencing perception and responses to uncertain stimuli. Because these moderators suggest that UT is contextual, the methodology and theoretical lens used for this review are a state-focused, qualitative approach wherein the interpretivist paradigm is adopted.

### Uncertainty tolerance conceptual model

In developing the IMUT, Hillen and colleagues (2017) explored the causes, effects, and nature of UT in a variety of contexts and describe three principal sources (stimuli) of healthcare-related uncertainty: probability; ambiguity; and complexity. The IMUT proposes that responses to uncertain stimuli can result in a spectrum of psychological responses from negative through to positive within three categories: cognitive, emotional, and behavioral. Within the cognitive category, the “thoughts” that are involved in response to perceiving uncertainty include examples like problem-solving in the presence of the uncertainty stimulus (Knight et al., 2016). The emotional category refers to the *feelings* that the individual provider encounters, when faced with perceived uncertainty such as strong emotional responses like anger (Norton, 1975 as cited in Hillen et al., 2017) or enjoyment (Budner, 1962 as cited in Hillen et al., 2017). The behavioral category explores the *actions* taken by providers in the face of uncertainty; behavioral responses range from adaptive responses such as judicious decision-making, to maladaptive responses such as resignation (i.e., giving up) on the negative end (Gerrity et al., 1990 as cited in Hillen et al., 2017). Based on the range of responses observed in the cognitive, emotional and behavioral categories, it is evident that aspects of responses to perceived uncertainty are individualistic and/or are contextually determined (Durrheim & Foster, 1997; Engelbrecht, 2000).

The IMUT expands on UT models by defining “moderators”, or factors that may influence an individuals’ perception of uncertainty and/or their responses to uncertainty (Hillen et al., 2017), suggesting that the construct could be state-based and contextually sensitive. In addition to providing broad categories of moderators, the IMUT illustrates where moderators act within the model, with suggestions that they exert effects in both the perception and response phases. This model, therefore, aids researchers by identifying a starting place for more specific characterization of healthcare-related moderators, extending the original broad moderator categories.

### Moderators of healthcare uncertainty

The presence of healthcare-related moderators of UT is a valuable area to explore further to improve healthcare provision. If moderators that have the potential to alter healthcare providers’ perception and responses to uncertainty can be comprehensively identified, this can be used to design interventions supporting healthcare providers in effectively managing uncertainty; potentially leading to improved patient-care outcomes, physician well-being and reductions in healthcare costs (Grutters et al., 2015; Hancock & Mattick, 2020; Jerak-Zuiderent, 2012a, 2012b). Inclusion of moderators in the IMUT suggests that an individual’s UT *is* susceptible to modification, at least in part. Based on the IMUT, a moderator may influence individuals, at two stages. The initial stage of influence occurs when individuals first encounter the uncertainty stimulus, and then after individuals perceive the uncertainty, influencing individuals’ responses. Moderators included in the IMUT are only described as broadly defined categories: Stimulus Characteristics; Individual Characteristics; Situational Characteristics; Cultural Factors; and Social Factors. Despite the potential importance of these moderators, Hillen et al. (2017) provides limited description of, or evidence for, these moderators.

Some recent work examining healthcare-related moderators identified certain incongruencies (Strout et al., 2018) wherein certain moderators (such as the level of experience in the role) reportedly had a positive impact in a given population, and in other populations the same moderator had either no impact or a negative impact (DeForge & Sobal, 1991; Geller et al., 1990; Lally & Cantillon, 2014; Merrill et al., 1994; Nevalainen et al., 2014). These incongruencies are reported in systematic reviews and meta-analyses of quantitative measures of UT, where exploration of the complex nature of moderators’ impacts on UT is limited due to the reliance on scale data wherein the multifaceted UT construct is defined by a singular number.

### Study context

The objective of this systematic review is to further define and explore healthcare-related moderators impacting providers’ perceptions and responses to uncertainty stimuli within this context. This systematic review serves to explore the evidence of moderators through the theoretical lens of the modern and comprehensive IMUT. Our research question was: ‘*What moderators of healthcare professionals’ tolerance of uncertainty within the clinical context are currently described, and how are they defined?’*, with our research aims focusing on; 1) further characterization of moderators of healthcare UT (Hillen et al., 2017); and 2) identify moderators impacts on the Cognitive, Emotional and Behavioral responses to uncertainty in healthcare professionals.

Given the contextual nature of moderators, this review uses an interpretivist paradigm. Supporting this paradigm, we explore qualitative literature in order to capture and identify moderator characteristics from healthcare professionals’ perspectives to address the moderator definitional gaps. Moderators are identified in this literature through healthcare providers’ (i.e., participant) descriptions of factors which impacted the participants’ perception(s) and/or response(s) to an identified healthcare uncertainty stimulus.

## Methods

### Search strategy

To explore definitional characteristics and categories of moderators of healthcare professionals’ UT, our research protocol followed the stepwise approach described by Cook and West (2012) and the PRISMA guidelines (Moher et al., 2009). Due to the currently broad generalizations characterizing moderators as they relate to healthcare UT in the IMUT, our search terms were purposely broad, and pertained to the UT construct and the healthcare context. The databases searched in this review were OvidMedline and Pubmed, CINAHL Plus, Embase, PsychINFO and Web of Science up to October 25, 2021. Our search strategy contained key words and synonyms of: tolerance or intolerance; uncertainty or uncertainties; ambiguity or ambiguities; health care or healthcare. For inclusion, studies needed to include at least one description of a healthcare providers’ “perception” or “response” when faced with ambiguity or uncertainty, as this helped define the potential moderators of UT. For this review only primary peer-reviewed research articles and research theses with qualitative research were included. As this work involved investigation of moderators of uncertainty tolerance, which is considered a complex phenomenon (Hillen et al., 2017) only articles published in English were included, this was to avoid any misunderstanding or misinterpretation of qualitative data in translated texts.

The literature search was conducted by one author (AY) with the assistance of a medical subject librarian (see acknowledgements). The electronic search strategy for each database is detailed in Table 1. For inclusion, studies needed to report primary accounts of participant’s descriptions of their experiences with uncertainty. This was particularly important as moderators were identified by analyzing and interpreting participant descriptions of factors which influenced the participants’ perception(s) and/or response(s) to an identified healthcare uncertainty stimulus, aligning with the principles of social constructionism where knowledge is constructed through joint experiences of individuals (Andrews, 2012; Galbin, 2014). Search results from each database were exported into EndNote™ (Clarivate Analytics, Philadelphia, PA), and duplicates were removed. Next, articles were exported into Microsoft Excel® (Microsoft Corporation) for the research team to keep track of how many articles were being screened at each stage, as well as reasons for exclusion.

**Table 1:** Electronic search strategie

Ovid medline and pubmed search strategy:	
#1 Search (uncertainty or uncertainties).mp. or UNCERTAINTY/	
#2 Search (ambiguity or ambiguities).mp. or ambiguity/	
#3 Search #1 or #2	
#4 Search (tolerance or intolerance).mp. [mp = title, abstract, original title, name of substance word, subject heading word, floating sub-heading word, keyword heading word, protocol supplementary concept word, rare disease supplementary concept word, unique identifier, synonyms]	
#5 Search exp medicine/	
#6 Search exp physicians/	
#7 Search healthcare.mp. or "Delivery of Healthcare"/	
#8 Search (medical or medicine or doctor* or physician* or GP or general practitioner* or surgeon* or resident* or nurs* or clinician* or clinical or patient care team).mp. [mp = title, abstract, original title, name of substance word, subject heading word, floating sub-heading word, keyword heading word, protocol supplementary concept word, rare disease supplementary concept word, unique identifier, synonyms]	
#9 Search #5 or #6 or #7 or #8	
#10 Search #3 and #4 and #9	
CINAHL Plus search strategy:	
#1 Search ambiguity or ambiguities	
#2 Search uncertainty or uncertainties	
#3 Search #1 or #2	
#4 Search tolerance or intolerance	
#5 Search #3 and #4	
#6 Search healthcare or healthcare	
#7 Search #5 and #6	
Embase search strategy:	
#1 Search (uncertainty or uncertainties).mp. or UNCERTAINTY/	
#2 Search (ambiguity or ambiguities).mp. or ambiguity/	
#3 Search #1 or #2	
#4 Search (tolerance or intolerance).mp. [mp = title, abstract, heading word, drug trade name, original title, device manufacturer, drug manufacturer, device trade name, keyword, floating subheading word, candidate term word]	
#5 Search exp medicine/	
#6 Search healthcare.mp. or exp healthcare/	
#7 Search (medical or medicine or doctor* or physician* or GP or general practitioner* or surgeon* or resident* or nurs* or clinician* or clinical or patient care team).mp	
#8 Search 5 or #6 or #7	
#9 Search #3 and #4 and #8	
PsychINFO search strategy:	
#1 Search (uncertainty or uncertainties).mp. or UNCERTAINTY/	
#2 Search STIMULUS AMBIGUITY/ or (ambiguity or ambiguities).mp	
#3 Search #1 or #2	
#4 Search (tolerance or intolerance).mp. or exp tolerance/	
#5 Search exp medical sciences/	
#6 Search (medical residency or medical students).mp	
#7 Search exp physicians/	
#8 Search (medical or medicine or doctor* or physician* or GP or general practitioner* or surgeon* or resident* or nurs* or clinician* or clinical or patient care team).mp	
#9 Search #5 or #6 or #7 or #8	
#10 Search #3 and #4 and #9	
Web of Science search strategy:	
TOPIC: ((ambiguity or uncertainty) AND (tolerance OR intolerance) AND (healthcare or medicine or medical science or medical or medicine or doctor* or physician* or GP or general practitioner* or surgeon* or resident* or nurs* or clinician* or clinical or patient care team*))	
Refined by: TOPIC	
Timespan = All years	
Search language = Auto	

### Data extraction and screening

**Fig. 1:**
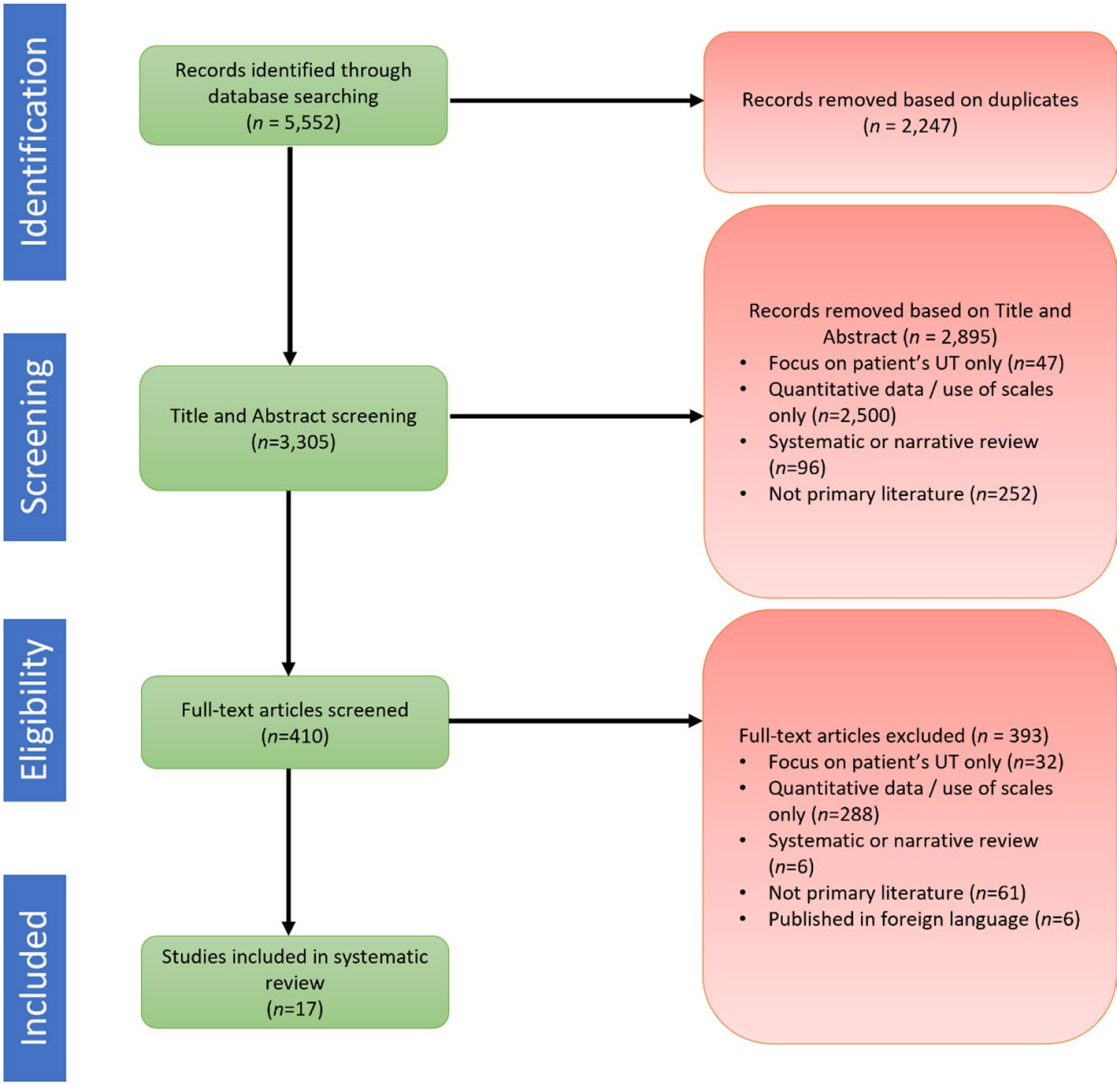
Summary of screening process and outcomes 5,552 articles were identified from our initial search strategy, post-duplicate removal, there were 3,305 articles that underwent title and abstract screening. Of these, 410 articles were identified for full-text screening and 393 articles (95.9%) were excluded due to not meeting the inclusion criteria, leaving 17 articles (4.1%) to be included in our systematic review

The screening process is outlined in Fig. 1 and was conducted in accordance with PRISMA guidelines (Moher et al., 2009). During the abstract screening stage, if the abstract met the inclusion criteria or if it was ambiguous, the article progressed to full-text screening stage. During the full-text screening stage, articles were included if the article met the established inclusion criteria (Table 2).

**Table 2:** Inclusion criteria

*Did the study explore one or more of the following concepts?*	
Tolerance or tolerance of uncertainty	
Tolerance or intolerance of ambiguity	
*Did the study explore the following healthcare populations?*	
Surgeons	
Physicians	
General practitioners	
Midwives	
Nurses	
Medical students on clinical placements	
Other healthcare providers (e.g. physiotherapist, psychologist, psychoanalysts)	
*Did the study include qualitative research on UT?*	
*The healthcare providers’ UT is impacted*	

Articles were excluded if they were only published in conference abstract form, grey literature, editorials, review articles, commentaries and books

Research data was extracted from the results section in the included articles by first author, and included details on the study design, study objective, data collection method and where possible, demographic data such as location, healthcare setting and healthcare profession. All extracted data was checked for accuracy by all authors. Post data extraction, and inclusion articles identified, The Critical Appraisal Skills Programme (2022) (CASP) Qualitative Research Checklist was used to appraise the quality of articles.

### Quality appraisal

The CASP checklist appraises the strengths and limitations of qualitative research by asking ten questions focusing on different methodological aspects within a qualitative study (Long, French & Brooks, 2020). The CASP checklist requires an answer of “yes”, “no” or “can’t tell” for each of the ten questions. “Yes” and “no” refers to whether or not the particular question is answered clearly, whereas “can’t tell” is used when it is unclear whether the question can be answered with a yes or no based on the information presented in a given study. The CASP checklist was used to independently appraise all included articles. On comparison of appraisals a final decision for each article was achieved through discussion to reach consensus.

### Data analysis

Framework analysis of the included articles was initially undertaken by author AY, with later stages incorporating PJ and CW. Framework analysis involved five stages: 1) familiarization; 2) identifying a thematic framework; 3) indexing; 4) charting and 5) mapping and interpretation (Ritchie & Spencer, 1994). Familiarization included scanning manuscripts for moderators. Based on this data several themes emerged and a thematic framework was developed. This framework was then used for coding at the indexing stage. During the indexing stage, initial codes, engaging a deductive approach wherein codes were explored for relationship to the existing IMUT. These codes were organized into themes and subthemes within each of the identified domains. The codes identified during the indexing stage were summarized with descriptions for each theme and subtheme. The descriptions for each theme into NVivo qualitative data analysis software, version 12.0 (QSR International, Melbourne, Australia) to assist with data organization. In the final stage, mapping and interpretation of the data, articles were coded by attributing quotations (phrases or sentences) to their relevant themes and subthemes into NVivo, and higher order relationships between codes were identified. Coding was checked through discussions with the manuscript authors.

To limit potential ambiguity in identification of stimuli vs. moderator vs. responses within the data, the following definitions were utilized based on the study participants point-of-view. Sources of uncertainty were defined as anything that stimulated participant uncertainty, while moderators were identified as downstream to these sources of uncertainty and also were reported as influencing participants’ perceptions and/or responses to the perceived uncertainty (stimulus). Downstream of these moderators is the uncertainty responses, referring to the behavioral, emotional, and cognitive outcomes as a result of the perceived uncertainty stimulus and the moderator influencing the response of the individual. All categories were identified through framework analysis of the participants’ description of the healthcare related uncertainty.

## Results

As illustrated in Fig. 1, the initial search identified 5,552 articles, of which 17 articles were included for analysis following full-text screening (Fig. 1 and Table 3). The CASP checklist indicated that the 17 included articles were of good to excellent quality (supplementary Table 1). A majority of the articles performed well on 80% of the criteria, in particular these articles scored well on the aims of research being clear (Q1), the use of appropriate qualitative methodology (Q2 and Q3), recruitment strategy (Q4) and data collection method (Q5), ethical considerations (Q7) as well as the findings were clearly reported (Q9) with consideration of contributions the field (Q10). The two criterion that scored “can’t tell” was the relationship between the researcher and participant (Q6) and whether the data analysis was rigorous (Q8). Given that none of the 17 articles had a “no”, and a majority of “yes”, this indicated that the included articles were of good to excellent quality.

**Table 3:** Summary of studies reviewed relating to factors impacting on the decision-making process of healthcare professionals (*N* = 17)

Author(s), Year	Objectives	Sample size (*N*) and method	Key themes and subthemes of identified moderators
Andre et al. (2016)	To investigate strategies for coping with uncertainty in relation to pharyngotonsillitis	*N* = 25 Method: Interviews	Presentation complexity Professionals’ concerns about repercussions
Borg et al. (2010)	To explore the concept of uncertainty tolerance in relation to crisis resolution and home treatment teams	*N* = unknown Method: Multistage focus group interviews	Uncertainty as it relates to professional identity (PI) and professional role (PR)
Bouchard (2016)	To explore the relationship between clinical uncertainty and compassion fatigue in nurses	*N* = 6 Method: Focus group interviews	Intrinsic uncertainty tolerance Presentation complexity
Causey Jr (2011)	To explore how experienced psychotherapists and psychoanalysts managed uncertainty during their sessions with narcissistically disordered patients	*N* = 5 Method: In-depth interviews	Uncertainty as it relates to professional identity (PI) and professional role (PR)Intrinsic uncertainty tolerance
Dogra et al. (2007)	To investigate the role of uncertainty in the teaching and learning of cultural diversity in medical students	*N* = 61 Method: Semi-structured interviews	Uncertainty as it relates to professional identity (PI) and professional role (PR)Skillset → Cultural competence
Fackler et al. (2009)	To examine the use of Cognitive Task Analysis (CTA) to analyse physician-team task analysis	*N* = *20* [14 doctors, 6 nurses] Method: Field observations and semi-structured interviews based on CTA	Intrinsic uncertainty tolerance Presentation complexity Role ambiguity Uncertainty as it relates to professional identity (PI) and professional role (PR)
Gowda et al. (2018)	To help students explore experiences of uncertainty and how to manage this through reflective practice using visual art	*N* = 47 Method: Group interviews and narrative post course evaluations	Uncertainty as it relates to professional identity (PI) and professional role (PR)Intrinsic uncertainty tolerance
Ilgen et al. (2020)	To explore how experienced clinicals respond to discomfort caused by uncertainty	*N* = *12* Method: Semi-structured interviews	Presentation complexity
Kenen et al. (2011)	To understand how healthcare professionals manage uncertainty when counseling and treating women with breast/ovarian cancer	*N* = 12 Method: Semi-structured interviews	Others’ perceptions Repercussions Uncertainty as it relates to professional identity (PI) and professional role (PR)Presentation complexity Collaboration → Teamwork
Knight et al. (2016)	To investigate the nursing characteristics and capabilities required to work in a rural setting	*N* = unknown Method: semi-structured interviews	Uncertainty as it relates to professional identity (PI) and professional role (PR)Holistic patient knowledge Skillset → Level of background or experience Presentation complexity
Morgan et al. (2007)	To examine the influences on GPs’ tendency to refer patients with headaches in the absence of clinical indicators	*N* = unknown Method: Qualitative interviews	Uncertainty as it relates to professional identity (PI) and professional role (PR)Presentation complexity Repercussions Personality characteristic → Level of self-confidence Skillset → Cultural competence Others’ perceptions
Nevalainen et al. (2009)	To investigate how medical students experienced uncertainty during their first year of clinical rounds	*N* = 22 Method: Reflective learning diaries	Repercussions Intrinsic uncertainty tolerance
Nurse-Clarke (2021)	To explore the experiences of nurses who care for women who experience stillbirth	*N* = *20* Method: Semi-structured interviews	Presentation complexity Collaboration > shared experiences and ownership
Page and Mander (2014)	To explore midwives’ perceptions of uncertainty when caring for women in low risk labor	*N* = 19 Method: Unstructured interviews and focus groups	Presentation complexity Repercussions Collaboration → Teamwork Collaboration → Shared experiences and ownership Personality characteristic → Level of self-confidence
Persson et al. (2011)	To explore how obstetricians managed pregnant women with gestational diabetes mellitus and uncertainty	*N* = 17 Method: Interviews	Collaboration → Shared experiences and ownership Collaboration → Teamwork Skillset → Level of background or experience
Roeske (2013)	To explore and analyse how experienced psychotherapists managed uncertainty in their clinical work	*N* = 8 Method: Semi-structured interviews	Uncertainty as it relates to professional identity (PI) and professional role (PR)Intrinsic uncertainty tolerance Others’ perceptions Personality characteristic → personal need for structure
van Iersel et al. (2019)	To explore how residents respond to clinical uncertainty when caring for geriatric patients	*N* = 9 Method: Semi-structured interviews	Collaboration → Shared experiences and ownership Collaboration → Teamwork Background or experience

**Fig. 2:**
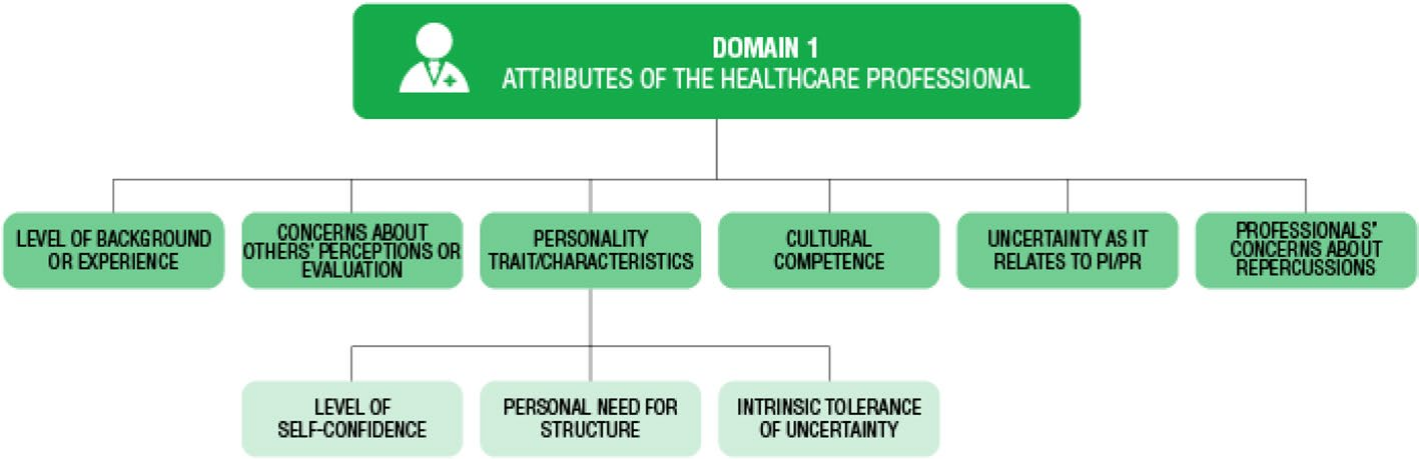
Domain 1: Attributes of the healthcare professional themes and subthemes. The identified themes were level of background or experience, concerns about others’ perceptions or evaluation, personality trait/ characteristics, cultural competence, uncertainty as it relates to PI and PR and professionals’ concerns about repercussions. Only one theme, personality trait/characteristics had subthemes: level of self-confidence, personal need for structure and intrinsic uncertainty tolerance

**Fig. 3:**
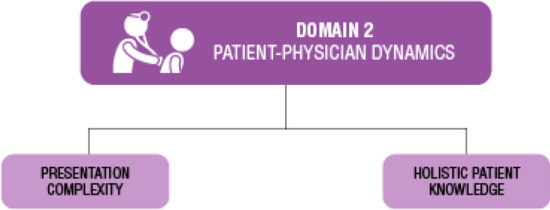
Domain 2: Patient-physician dynamics themes. Two themes were identified; presentation complexity and holistic patient knowledge

**Fig. 4:**
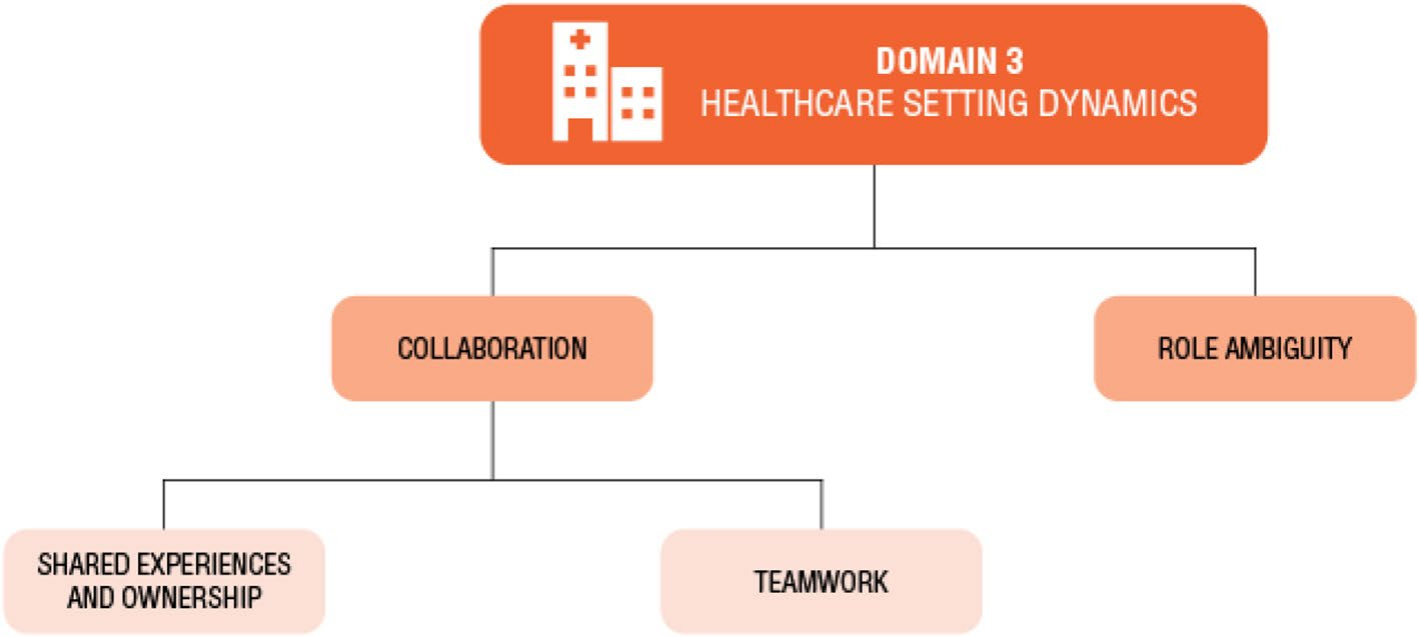
Domain 3: Healthcare setting dynamics themes and subthemes. Two themes were identified within this domain; role ambiguity, and collaboration. Under the theme of collaboration, two subthemes were identified: shared experiences and ownership, and teamwork

Through the use of inductive coding, three domains moderating healthcare professionals’ uncertainty tolerance were identified. These included: Domain 1: Attributes of the healthcare professional; Domain 2: Patient-physician dynamics; and Domain 3: Healthcare setting dynamics. Each domain included multiple themes, with many containing subthemes (Figs. 2, 3 and 4). The domains, their associated themes and subthemes, are described below. Within each theme and subtheme, moderators were identified when study participant’s descriptions of this factor appeared to influence their perception and/or response(s) to UT. An outline of the moderators and their influences on healthcare providers’ reported responses to uncertainty identified in this analysis are summarized in Table 4.

**Table 4:** Moderators and responses to uncertainty

			Response	
Domain	Moderator	Cognitive	Emotional	Behavioral
Domain 1: Attributes of the healthcare professional	Level of background or experience	Problem-solve (Knight et al., 2016) Tolerance to ambiguous stimuli (Kenen et al., 2011; Page & Mander, 2014) Decision-making (Knight et al., 2016)	Unknown	Refer patient (Morgan et al., 2007; Persson et al., 2011)
	Concerns about other’s perceptions or evaluation		Frustration towards uncertainty (Kenen et al., 2011) Unease/Discomfort (Roeske, 2013)	Non-disclosure (Morgan et al., 2007)
	Personality characteristic > Level of self-confidence	Aversion to ambiguity (Morgan et al., 2007) Decision-making (Morgan et al., 2007; Page & Mander, 2014)	Anxious (Morgan et al., 2007)	Refer patient (Morgan et al., 2007) Inaction (Morgan et al., 2007; Page & Mander, 2014)
	Personality characteristic > Personal need for structure	Tolerance to ambiguous stimuli (Page & Mander, 2014) Aversion to ambiguity (Roeske, 2013) Confusion at ambiguous stimuli (Roeske, 2013)	Unease/Discomfort (Roeske, 2013)	Inaction (Roeske, 2013)
	Personality characteristic > Intrinsic uncertainty tolerance	Unknown	Unease/Discomfort (Dogra et al., 2007) Anxious (Dogra et al., 2007) Cautious (Dogra et al., 2007; Fackler et al., 2009)	Refer patient (Morgan et al., 2007)
	Professionals’ concerns about repercussions	Aversion to ambiguity (Kenen et al., 2011; Nevalainen et al., 2009; Page & Mander, 2014)	Unease/Discomfort (Morgan et al., 2007; Nevalainen et al., 2009)	Refer patient (Morgan et al., 2007)
	Cultural competence	Unknown	Unease/Discomfort (Morgan et al., 2007)	Refer patient (Morgan et al., 2007)
	Uncertainty as it relates to professional identity (PI) and professional role (PR)	Acknowledgement of uncertainty (Dogra et al., 2007; Kenen et al., 2011; Knight et al., 2016) Acceptance or ownership of uncertainty (Kenen et al., 2011; Knight et al., 2016)	Anger (Causey Jr, 2011) Unease/Discomfort (Gowda et al., 2018; Morgan et al., 2007) Fear of failure (Page & Mander, 2014)	Unknown
Domain 2: Patient-physician dynamics	Presentation complexity	Confusion at ambiguous stimuli (Andre et al., 2016; Bouchard, 2016; Fackler et al., 2009; Ilgen et al., 2020; Page & Mander, 2014) Acknowledgement of uncertainty (Page & Mander, 2014) Problem-solve (Andre et al., 2016; Fackler et al., 2009) Intolerance (Andre et al., 2016; Fackler et al., 2009; Morgan et al., 2007)	Anxious (Nurse-Clarke, 2021)	Refer patient (Ilgen et al., 2020; Morgan et al., 2007) Inaction (Andre et al., 2016; Page & Mander, 2014) Avoid patient (Nurse-Clarke, 2021)
	Holistic patient knowledge	Problem-solve (Knight et al., 2016)	Unknown	Unknown
Domain 3: Healthcare setting dynamics	Collaboration > shared experiences and ownership	Tolerance to ambiguous stimuli (Ilgen et al., 2020; Page & Mander, 2014; Persson et al., 2011; van Iersel et al., 2019)	Unknown	Unknown
	Collaboration > teamwork	Tolerance to ambiguous stimuli (Page & Mander, 2014) Decision-making (Kenen et al., 2011; Page & Mander, 2014; Persson et al., 2011)	Unknown	Collaborative care management (Kenen et al., 2011)
	Role ambiguity	Confusion at ambiguous stimuli (Fackler et al., 2009; Page & Mander, 2014)	Unknown	Inaction (Fackler et al., 2009; Page & Mander, 2014)

The following cognitive outcomes are identified as positive: problem-solving, tolerance to ambiguous stimuli, decision-making, ownership of uncertainty. No positive emotional responses are identified and/or were reported. The positive behavioral responses identified included: resisting patient behavior and collaborative care management

### Domain 1: Attributes of the healthcare professional

The attributes of the healthcare professional domain encapsulate moderators identified as affecting the characteristic patterns of thinking, feelings, skills, or experiences inherent and unique to an individual provider. Under this domain, multiple themes and subthemes were identified (Fig. 2).

#### Background or experience

The theme background or experience was identified in six articles describing individuals’ experience levels influencing providers’ UT (Kenen et al., 2011; Knight et al., 2016; Morgan et al., 2007; Page & Mander, 2014; Persson et al., 2011; van Iersel et al., 2019). Participant descriptions related to this code indicate that this moderator appears to predominantly affect cognitive responses to uncertainty stimuli, particularly as it relates to providers’ decision-making processes (Table 4). This moderator appears to result in negative modulation of the providers’ behavioral response resulting in patient referral (Table 5) (Morgan et al., 2007; Persson et al., 2011). However, providers’ prior experiences are the prevailing basis for positively affecting problem-solving capacity within the complex healthcare environment (described as decision-making in the integrative model yielding a positive cognitive response to the uncertainty stimuli (Hillen et al., 2017)). Conversely, a lack of experience appears to impede a healthcare providers’ ability to address clinical difficulties, leading to Hillen’s (2017) described “inaction” or negative uncertainty tolerance as a behavioral response. Whilst background or experience appears to assist individuals with making informed decisions (i.e., behaviorally tolerant of ambiguity), the literature included in this review suggests that experience may not always be a positive moderator of clinicians’ uncertainty tolerance. Reported negative clinical experiences, for instance a provider previously missing a diagnosis (Morgan et al., 2007), results in adverse behavioral effects in the clinical decision-making process with a suggested response of providers’ delaying decision-making or indecision (behaviorally less tolerant of uncertainty based on the IMUT).

**Table 5:**
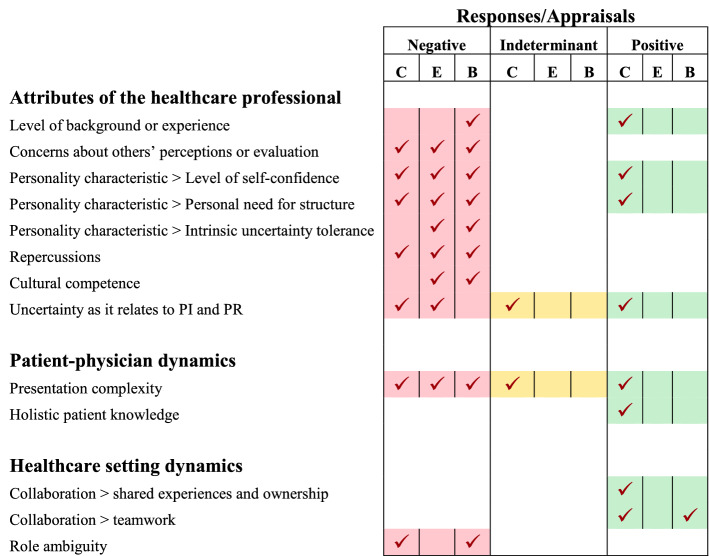
Summary of appraisals for each subtheme related to the three identified domains; *D*1 Attributes of the healthcare professional; *D*2 Patient-physician dynamics; and *D*3 Healthcare setting dynamics. The shading reflects the negative (red), indeterminant (yellow) and/or positive (green) appraisals and/or responses observed in the literature for each of the given subthemes. *C* = cognitive, *E* = emotional, *B* = behavioral. Negative and positive responses were based on the IMUT (Hillen et al., 2017). Indeterminant indicates when the moderator influenced the healthcare provider, but it was unclear whether the influence on providers’ responses to uncertainty were positive or negative valency

#### Concerns about other’s perceptions or evaluation

Another moderator of the healthcare professional’s UT emerges as concerns about other’s perceptions or evaluation of their clinical expertise. This moderator was identified in three articles and potentially influences behavior during interactions with patients (Kenen et al., 2011; Morgan et al., 2007; Roeske, 2013). For example, the providers’ concerns about how others will perceive or evaluate their clinical expertise when divulging uncertainty can lead to some providers not divulging uncertainty. Articles describing this moderator discuss a perception in the healthcare field that sharing a lack of knowledge in the face of clinical ambiguity is indicative of professional incompetence (Kenen et al., 2011; Morgan et al., 2007; Roeske, 2013). From the three articles, the moderator of ‘concerns about other’s perceptions or evaluation’ is predominantly derived from patients’ perceptions of the clinician, and not peers’ perceptions.

Participant discussion suggests that moderator influences the cognitive, emotional and behavioral responses (Table 5), resulting in a lack of disclosure of uncertainty to patients. Our review of the literature suggests that this altered communication is moderated by the clinician's internalization of others' perceptions of them (Kenen et al., 2011; Morgan et al., 2007; Roeske, 2013). This moderation appears to result from a belief that disclosing uncertainty, or “not knowing”, would indicate a lack of experience, self-confidence, and competence within their professional role.

#### Personality characteristics

A personality characteristic was defined as an individuals’ thoughts, feelings and behaviors. These can be intrinsic to them or developed over time (Allport, 1929). Three subthemes were identified in this theme: level of self-confidence, personal need for structure and intrinsic uncertainty tolerance.

##### Level of self-confidence

Self-confidence was considered as a personality characteristic based on the nature in which individuals reported on their own self-confidence in relation to healthcare uncertainty in two articles (Morgan et al., 2007; Page & Mander, 2014). Since, in this context, self-confidence is measured by self-reporting, then it was considered a personality characteristic (Burns et al., 2016). Behavioral outcomes including disclosure of uncertainty, referring patients and patient behavioral management all appear to be moderated, at least in part, by provider self-confidence. In the IMUT, personal characteristics are included as a moderator of UT. The providers’ level of self-confidence (a ‘personal characteristic’) appears to have some influence on providers’ responses to healthcare uncertainty stimuli (Table 5). The moderator of self-confidence appears to influence behavioral responses, including managing patient behaviors perceived by the individual provider as challenging or difficult (Morgan et al., 2007). Individuals with lower clinical self-confidence appear more likely to refer patients to a specialist as opposed to managing the healthcare uncertainty themselves (Morgan et al., 2007), though the appropriateness of these referrals remains unclear.

In part, this lower clinical self-confidence may result in increased referrals due to fears of misdiagnosing. Articles identified in this systematic review suggest that the individual providers’ self-confidence level also moderates their cognitive and emotional responses. The providers’ response of patient referral appears linked to their lower clinical confidence, and thus moderates the providers’ perception of the uncertain stimulus behaviorally (Morgan et al., 2007; Page & Mander, 2014). This moderator, unlike many in this first domain, appears to influence cognitive responses in addition to behavioral responses, and may manifest as aversion to the ambiguous stimuli (Table 4) by some providers faced with presentation complexity (Morgan et al., 2007). This cognitive appraisal may intercalate with negative behavioral responses including expressed reluctance to clinically manage patients when dealing with perceived presentation complexity (i.e., deferral as classified by Hillen et al., 2017) and preferences to refer patients “just in case” of missing “something”.

##### Personal need for structure (PNS)

Personal need for structure (PNS) was identified in two articles (Page & Mander, 2014; Roeske, 2013) as a need or preference for structure which appears to affect providers’ responses/appraisals across multiple domains (Table 5). PNS is a theoretical construct suggesting that individuals can reduce uncertainty of a situation by having structure to mitigate uncertainty or ambiguity (Švecová & Pavlovičová, 2016). This PNS moderator was defined from literature highlighting individuals who perceived guidelines and protocols to be “correct” or the singular truth. Providers whose responses to uncertainty were moderated by a need for structure or to follow guidelines and/or protocols; this commitment to structure may be linked to a strong belief that adherence to structure would reduce inherent clinical uncertainty (Page & Mander, 2014; Roeske, 2013). As a moderator, PNS resulted in expressions of “confusion” when presented with a lack of structure and/or struggling with making sense of the uncertainty stimuli when structure was absent (cognitive and emotional) (Page & Mander, 2014; Roeske, 2013) (Table 4). Behaviorally, this moderator appears to influence individuals through a struggle to proceed with making clinical decision-making when guidelines were unavailable or if a situation did not strictly follow guidelines and/or procedures (Page & Mander, 2014; Roeske, 2013). PNS appears to result in lower clinician uncertainty tolerance as categorized by Hillen’s (2017) descriptions.

##### Intrinsic uncertainty tolerance

An individual’s intrinsic uncertainty tolerance was also identified as a subtheme in six articles (Bouchard, 2016; Causey Jr, 2011; Fackler et al., 2009; Gowda et al., 2018; Nevalainen et al., 2009; Roeske, 2013). This moderator was identified from data highlighting how providers’ perceptions of healthcare ambiguity was influenced by working with colleagues, as being part of a team allowed them to collectively manage the clinical situation stimulus (i.e., the ambiguous stimulus) (Bouchard, 2016; Causey Jr, 2011; Fackler et al., 2009; Gowda et al., 2018; Nevalainen et al., 2009; Roeske, 2013). The literature identifying this moderator predominantly highlights resultant negative uncertainty responses, as conceptualized in the IMUT. Cognitive appraisals, along with emotional and behavioral responses, all appear to be influenced by intrinsic uncertainty tolerance (Bouchard, 2016; Causey Jr, 2011; Fackler et al., 2009; Gowda et al., 2018; Morgan et al., 2007; Nevalainen et al., 2009; Roeske, 2013). In this context, negative cognitive appraisals moderated by intrinsic uncertainty tolerance are identified with providers discussing an inability to proceed with action regardless of how simple, complex, or ambiguous the clinical situation is (Fackler et al., 2009; Gowda et al., 2018; Roeske, 2013) (Table 5). Emotionally, this moderator may result in providers feeling *uneasy*, *anxious* and *cautious* when faced with uncertainty stimuli (Table 5). Providers’ behavioral responses to this moderator include consistent erring on the side of caution and referring patients when faced with clinical uncertainty (Table 4).

#### Cultural competence

Another personal characteristic moderator identified in two articles was cultural competence. Cultural competence is defined from descriptions of providers’ having (or not) the ability to communicate and understand people from various cultural backgrounds. This perceived lack of cultural competence could be as a result of cultural discomfort, in which cultural differences contribute to uncertainty and unease. Review of included literature specifies that cultural competence may influence providers’ emotional and behavioral responses to healthcare uncertainties (Dogra et al., 2007; Morgan et al., 2007). A lack of cultural competence results in reported emotional feelings of *uneasiness* when communicating, in general, with patients from a culture different to that of the provider) and a behavioral response of patient referral (Morgan et al., 2007). Together, this suggests that a lack of cultural competence moderates providers’ responses negatively. There are no reports identified within the papers included in our study, of positive appraisals/responses moderated by cultural competency (Table 5).

#### Uncertainty as it relates to professional identity (PI) and professional role (PR)

The final theme identified in the attributes of the healthcare provider domain explores the notion of uncertainty as it relates to professional identity (PI) and professional role (PR) in nine articles (Borg et al., 2010; Causey Jr, 2011; Dogra et al., 2007; Fackler et al., 2009 Gowda et al., 2018; Kenen et al., 2011; Knight et al., 2016; Morgan et al., 2007; Roeske, 2013). PI refers to the expectations of the healthcare provider (the self) in their role (Hendrikx, 2018) and professional role refers to the expected function (by others) of the providers’ role (Furåker, 2008). The uncertainty as it relates to PI/PR appears to influence clinicians’ emotional and cognitive responses (Causey Jr, 2011; Gowda et al., 2018; Morgan et al., 2007). Cognitive appraisals affected by this moderator include acknowledgement that uncertainty existed as part of their professional role and/or identity (Dogra et al., 2007; Kenen et al., 2011; Knight et al., 2016) and suggests a positive influence on healthcare uncertainty responses. Herein, individuals discussed the nature of their roles being inclusive of uncertainty (Borg et al., 2010; Causey Jr, 2011; Dogra et al., 2007; Gowda et al., 2018; Kenen et al., 2011; Knight et al., 2016; Morgan et al., 2007; Roeske, 2013). However, a spectrum of cognitive and emotional responses was identified; some individuals responded with acceptance of uncertainty being integral in the nature of their work (Kenen et al., 2011; Knight et al., 2016), even going so far as suggesting that this uncertainty somewhat improved their professional practice (positive response), whilst others expressed emotional “uneasiness” or “anger” towards uncertainty in their role (Causey Jr, 2011; Gowda et al., 2018; Morgan et al., 2007) indicative of Hillen’s (2017) described negative cognitive appraisals. No reporting of this moderator on behavioral responses was identified.

#### Professionals’ concerns about repercussions

The moderator theme professionals’ concerns about repercussions was identified in five articles where individual providers expressed concern or fear of making a mistake, and the resultant consequences of their behaviors or actions in relation to this concern (Andre et al., 2016; Kenen et al., 2011; Morgan et al., 2007; Nevalainen et al., 2009; Page & Mander, 2014). Professionals’ concerns about repercussions appears to negatively impact providers’ responses to uncertainty across all three IMUT categories (cognitive, emotional and behavioral). Individual providers were reportedly more likely to refer patients (behavioral) and delay decision-making, express an aversion to ambiguity (cognitive) (Kenen et al., 2011; Morgan et al., 2007), and feel uneasiness or discomfort in the presence of the perceived healthcare ambiguity (emotional).

Together, moderators that fall into the healthcare provider domain may influence the providers’ behavioral responses to healthcare uncertainty, although (to a lesser extent) cognitive and emotional responses are also identified (Table 5). While some moderators appear to affect providers’ perception of healthcare uncertainty positively, our data identified a majority of moderators within this domain in the negative category (Table 5).

### Domain 2: Patient-physician dynamics

This domain refers to moderators influenced by provider interactions with a patient. These moderators relate to the healthcare provider’s relationship with their patient in some capacity. Two themes were identified in this domain including presentation complexity and holistic patient knowledge (Fig. 3).

#### Presentation complexity

The moderator theme presentation complexity was identified in nine articles describing healthcare providers responses to uncertainty that were influenced by the spectrum of potential diagnoses related to patient symptoms or clinical presentations (i.e., the uncertain stimulus) (Andre et al., 2016; Bouchard, 2016; Fackler et al., 2009; Ilgen et al., 2020; Kenen et al., 2011; Knight et al., 2016; Morgan et al., 2007; Nurse-Clarke, 2021; Page & Mander, 2014). Herein, some providers noted inconsistencies between the presenting patients’ symptoms and the array of potential presentations for the particular condition or conditions. Presentation complexity is the only theme in Domain 2 to show an emotional response of feeling *anxious* due to presentation complexity (Nurse-Clarke, 2021). In this theme, cognitive appraisals are the predominant responses linked to this moderator, with reported expressions of “confusion” and/or “intolerance” of the ambiguous stimuli derived from presentation complexity (Andre et al., 2016; Fackler et al., 2009; Morgan et al., 2007; Page & Mander., 2014). This, for example, led to behavioral responses such as “referral” (Morgan et al., 2007) due to the cognitive appraisal of a lack of described “coping” with this atypical clinical uncertainty (Hillen et al., 2017). It is possible that presentation complexity acts to modulate cognitive appraisals, with some providers acknowledging that defining the boundaries of ‘normality’ results in acknowledgement of uncertainty in the patient presentation (Page & Mander, 2014), suggesting a moderating influence on providers’ appraisals of healthcare uncertainty.

#### Holistic patient knowledge

Relating to the patient, a provider’s holistic patient knowledge appears to moderate clinicians’ uncertainty tolerance in one article (Knight et al., 2016). Holistic patient knowledge refers to the provider’s experience as it relates to a known patient, separate and distinct from their accrued professional experience (in Domain 1). Here, the providers’ intimate and extensive knowledge of the individual patient is what appears to be moderating the healthcare providers’ uncertainty responses (Knight et al., 2016). Literature suggests that those with holistic patient knowledge are influenced in both their cognitive and behavioral responses (Table 4). In Knight et al. (2016), rural nurses appear to have improved problem-solving capacity (cognitive) when faced with complex clinical presentations, enabling them to make clinical decisions and act despite this uncertainty (Knight et al., 2016). This potentially indicates that this moderator may have a positive influence on providers’ perception and responses to healthcare uncertainty stimuli (Knight et al., 2016).

### Domain 3: Healthcare setting dynamics

This final domain was identified from six articles highlighting moderators of healthcare providers’ UT associated with the healthcare setting and infrastructure (Fackler et al., 2009; Kenen et al., 2011; Nurse-Clarke, 2021; Page & Mander, 2014; Persson et al., 2011; van Iersel et al., 2019); these moderators are inclusive of healthcare teams, resource availability and time pressures faced by healthcare providers (Fig. 4). This domain explores how these healthcare organizations, resources and environments moderate the individual providers’ uncertainty tolerance responses.

#### Collaboration

The theme collaboration was identified in articles referring to communication between individual providers and their teams which moderated healthcare providers’ perception of uncertainty. Two subthemes were identified: shared experiences and ownership; and teamwork.

##### Shared experiences and ownership

The moderator of shared experiences and ownership was defined from four articles describing collective experiences and related teamwork modulating healthcare providers’ responses to uncertainty (Nurse-Clarke, 2021; Page & Mander, 2014; Persson et al., 2011; van Iersel et al., 2019) A sense of shared responsibility appears to assist behavioral decision-making outcomes when faced with ambiguous healthcare stimuli (Nurse-Clarke, 2021; Page & Mander, 2014; Persson et al., 2011; van Iersel et al., 2019). This moderator manifests in the literature as collective team discussions focused on consensus building of patient management next steps (Page & Mander, 2014; Persson et al., 2011), and results in individuals feeling a decreased pressure to “get it right”. In this way, this moderator appears to positively affect uncertainty perceptions; as a result of this shared responsibility (moderator), providers report being able to make collective decisions (positive behavioral response), and limit perceived individual responsibility.

##### Teamwork

While shared responsibility assists with positively moderating behavioral responses, it is also evident that teamwork (identified in four articles) may play a role in moderating uncertainty by acting as a source of mutual education and support for coworkers (Kenen et al., 2011; Page & Mander, 2014; Persson et al., 2011; van Iersel et al., 2019). In this theme, when individuals consulted their coworkers, there was a positive cognitive response whereby individuals demonstrate that they are tolerant of ambiguity (Kenen et al., 2011; Page & Mander, 2014; Persson et al., 2011; van Iersel et al., 2019).

#### Role ambiguity

The final theme, role ambiguity, was identified in two articles where individuals described unclear professional role boundaries and/or guidelines influencing the individual providers’ responses to uncertainty (Fackler et al., 2009; Page & Mander, 2014). In this theme, individuals experience a cognitive response such as “confusion” related to their roles and responsibilities within the healthcare setting (Fackler et al., 2009; Page & Mander, 2014), predominantly stemming from perceptions that their roles were ambiguously defined. These individuals express that a lack of clarity regarding the intersection of roles within their healthcare team (moderator) influences their uncertainty in the healthcare setting (cognitive appraisal). This not only appears to affect their cognition relating to healthcare uncertainty, but also may result in behavioral response modifications as well. For example, healthcare professionals appear to struggle with inaction when feeling uncertain about their role in a clinical case or clinical cases (Fackler et al., 2009; Page & Mander, 2014) (Table 4). Literature which highlights this moderator of role ambiguity suggests that it predominately moderates providers’ uncertainty responses negatively (Table 4), as no reports of positive responses were identified (Table 5).

## Discussion

The aim of this systematic review was to further characterize moderators of UT in healthcare, to expand on, and further refine, the moderator component of the IMUT by Hillen et al. (2017). Despite the great potential of the moderator component of the IMUT to influence providers’ UT, the model lacks sufficient detail. While the IMUT proposes moderators as influencers of healthcare providers’ perceptions and responses to uncertainty, there are significant gaps in regarding the nature, and impact, of these moderators. Within the IMUT, moderators were broadly identified as “Stimulus Characteristics, Individual Characteristics, Situational Characteristics, Cultural Factors and Social Factors” (Hillen et al., 2017). This systematic review’s findings suggest moderators have the potential to influence healthcare providers’ UT and provide a potential avenue for training medical students, junior doctors or other healthcare professionals.

### Extending the IMUT: characterizing moderators

**Fig. 5:**
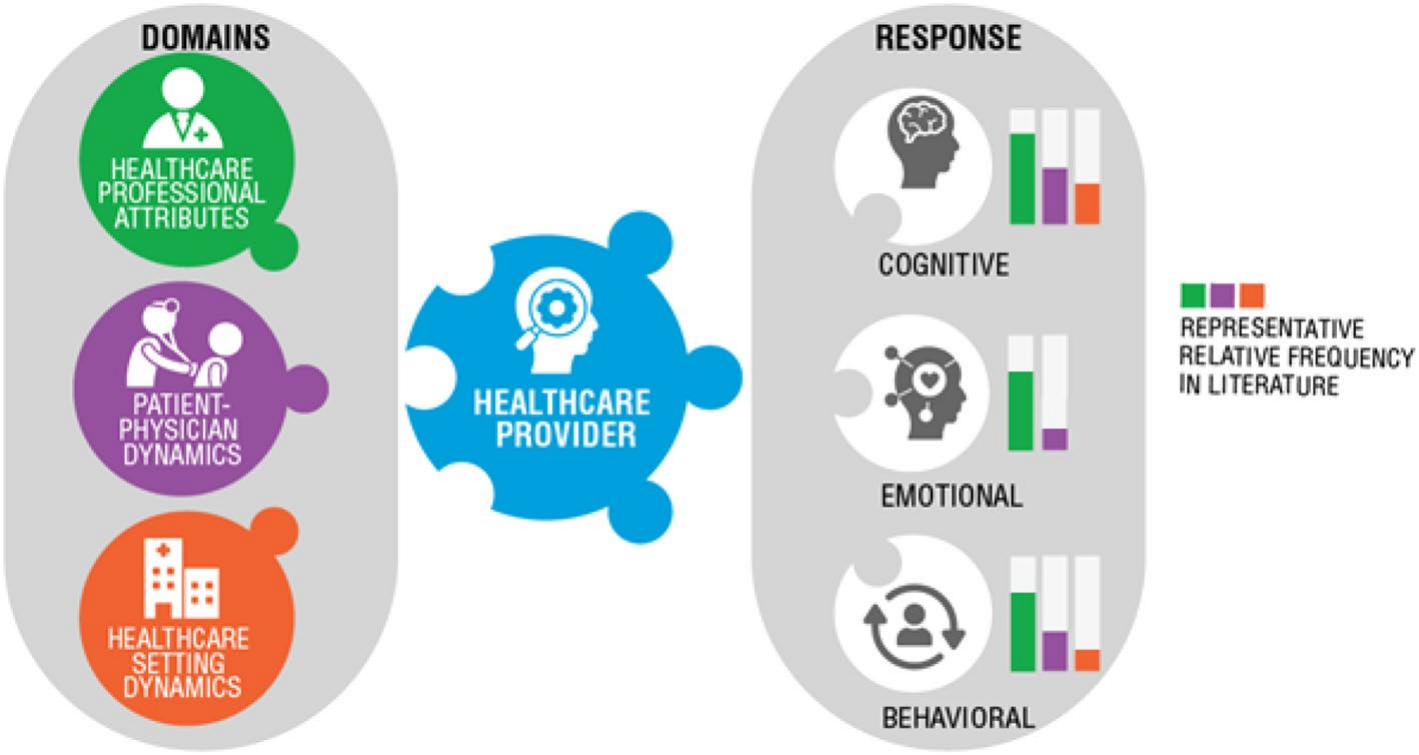
Influences of moderators in healthcare professionals and their cognitive, emotional and behavioral responses. *D*1 and *D*2 influences the healthcare providers’ perception and cause cognitive, emotional and behavioral responses, with a majority of *D*1 responses present in cognitive appraisals. *D*3 only influences the cognitive appraisal and behavioral responses of healthcare professionals

This systematic review extends the IMUT by supporting many of the broad categories of moderators and further extends these categories through its nuanced description of both the moderator definitions, and their relationship(s) to healthcare providers’ perceptions and/or responses across the three categories (cognitive, emotional and behavioral) (Fig. 5).

The moderators that fall into Domain 1 are most closely linked to the IMUT moderator category of Individual Characteristics (Table 6), referring to the intrinsic personality and nature of the healthcare professional themselves. These moderators appear to generously influence the cognitive, emotional, and behavioral responses of providers faced with healthcare uncertainty (Fig. 5, green), although cognitive appraisals and emotional responses were also linked to Domain 1 moderators. While some moderators appear to influence providers’ responses to healthcare uncertainty positively, this systematic review found that the majority of moderators within this domain are reportedly associated with negative appraisals and responses (Table 5) (Causey Jr, 2011; Dogra et al., 2007; Fackler et al., 2009; Kenen et al., 2011; Knight et al., 2016; Page & Mander, 2014; Persson et al., 2011; Roeske, 2013). This suggests that there may be opportunities to explore moderators of UT in healthcare providers for further insight as to whether these moderators have the potential to positively influence UT. By doing so, this may provide a more holistic understanding of how moderators in the Individual Characteristics category may foster or hinder UT, potentially enabling the field to identify specific methods to foster the changeable moderators within this category. While a majority of the moderators reported in this Domain are negative, it is possible that these studies could be biased towards reporting on factors that focus on the negative aspects of responses to uncertainty tolerance.

**Table 6:** Identified moderators of UT and their allocations in the integrative model

Theme	Subtheme	Integrative model (5 broad categories)
*D*1: Background or experience	N/A	Individual characteristics
*D*1: Concerns about other’s perceptions or evaluation	N/A	Individual characteristics
*D*1: Personality characteristic or characteristic	Level of self-confidence	Individual characteristics
*D*1: Personality characteristic or characteristic	Personal need for structure	Individual characteristics
*D*1: Personality characteristic or characteristic	Intrinsic uncertainty tolerance	Individual characteristics
*D*1: Cultural competence	N/A	Individual characteristics
*D*1: Uncertainty as it relates to PI and PR	N/A	Individual characteristics
*D*1: Repercussions	N/A	Individual characteristics
*D*2: Presentation complexity	N/A	Stimulus characteristics / Situational characteristics
*D*2: Holistic patient knowledge	N/A	Stimulus characteristics / Situational characteristics
*D*3: Collaboration	Shared experiences and ownership	Situational characteristics
*D*3: Collaboration	Teamwork	Situational characteristics
*D*3: Role ambiguity	N/A	Situational characteristics

A majority of *D*1 moderators fall into the Individual Characteristics category, while *D*2 moderators are more suitable for the Stimulus Characteristics and Situational Characteristics categories. In *D*3, the moderators appear to be in line with the Situational Characteristics category

The moderators identified in Domain 2, align with two broad categories, Stimulus Characteristics and Situational Characteristics (Table 6) with moderator themes encompassing presentation complexity and interactions with patients. Our findings suggest that moderators of uncertainty originating from the doctor-patient dynamic can influence responses to uncertainty within all three Hillen et al. (2017) response categories. Domain 2 moderators, however, appear to influence cognitive appraisals, with trends of cognitive “confusion” (Andre et al., 2016; Bouchard, 2016; Fackler et al., 2009; Ilgen et al., 2020; Page & Mander, 2014), suggesting that patient-related uncertainty tends to link with negative cognitive appraisals by healthcare providers (Table 4). Similar to Domain 1, a majority of the reported literature suggests that moderators in Domain 2 are mostly linked to negative responses with few reports reporting positive associations. These findings further contribute to the discussion regarding whether UT is context-specific and situational rather than their Individual Characteristics (as seen in Domain 1). This finding can be further explored to identify components that can be controlled in a contextual environment to reduce uncertainty stimulus.

Contrary to the mostly negative responses reported in the prior domains, Domain 3 moderators appear to positively influence participants’ responses in the face of healthcare uncertainty stimuli (Table 5). Moderators in this domain appear to fall into the broad moderator category of Situational Characteristics as defined by the IMUT (Table 6), specifically the moderators of collaboration and role ambiguity. The mostly reported positive responses in this domain suggests that working in teams is a successful moderator for managing uncertainty in healthcare providers. Thus, there may be opportunities for clinical teams to partner or cultivate an environment for shared decision-making to positively influence UT in individual providers.

### Impact of moderators on uncertainty tolerance

Our work further extends the IMUT by not only providing more detail about moderator-related characteristics, but also their potential influence and links to providers’ uncertainty perceptions and responses. Understanding the relationship between uncertainty perceptions and responses may assist with identifying potential training to positively influence uncertainty tolerance in healthcare providers. This systematic review identifies that some moderators (e.g., the level of background or experience, level of self-confidence, uncertainty as it relates to PI and PR, clinical presentation, holistic patient knowledge and teamwork) can result in a variety of responses from negative, indeterminant to positive responses to uncertainty. This supports previous inconclusive associations with uncertainty tolerance as it relates to age (Strout et al., 2018), experience (McCulloch et al., 2005; Nevalainen et al., 2014), and fear of making mistakes (Nevalainen et al., 2012, 2014). The results presented here begin to provide a basis for the apparent inconsistency of these moderators. For instance, healthcare experience (i.e., more clinical experience versus less clinical experience or more senior in medical education versus less senior) is shown to be variable in its effects on providers’ uncertainty tolerance (DeForge & Sobal, 1991; Merrill et al., 1994; Nevalainen et al., 2012; Weissenstein et al., 2014). This systematic review suggests that this variability may not necessarily be due to study population or survey instrument used, but rather to the types of experiences a provider has had to the point at which their UT is evaluated. Findings suggest that a healthcare provider with a great deal of experience, under normal conditions, will likely have a higher UT, while a provider who has had a negative patient outcome, regardless of experience level, may have a lower UT (Morgan et al., 2007; Page & Mander, 2014). This may also explain the previous inconsistent results in different populations (Strout et al., 2018), as it is not always clear which domain is being assessed in a given scale, and/or results are not separated into these three domains upon analysis. Our results suggest that moderators can influence cognitive, emotional and behavioral responses to uncertainty, and thus interpretation of scales in future may want to consider this by categorizing scale items based on the response domain being assessed.

### Potential impact on healthcare settings

The findings of this systematic review highlight moderators which may be modifiable or changeable within a healthcare setting. By focusing on adjustments to these particular moderators, healthcare systems could help foster uncertainty tolerance (as opposed to promoting or accepting *in*tolerance) with potential to ultimately improve patient outcomes and care given the strong links between healthcare and patient outcomes (Kim & Lee, 2018).

Moderators such as clinical self-confidence and cultural competence are previously shown to be positively influenced through various interventions. Kwiatkowski et al. (2014) found that medical students were able to increase their self-confidence to perform patient-care skills through early clinical immersion exposure. Herein, students presented with authentic scenarios encountered by emergency medical technicians (EMTs) would work through these scenarios to practice the necessary skills required in particular scenarios (Kwiatkowski et al., 2014). Results from these studies suggest that clinical self-confidence may be a modifiable trait, and that education may be a powerful tool in influencing this process.

Collaboration was also shown to positively influence healthcare uncertainty tolerance. Building positive relationships within interprofessional teams has positive influences on healthcare delivery teams (Bajnok et al., 2012). These influences include an increase in team function, work satisfaction levels, the ability to work independently and improved patient wellbeing (Bajnok et al., 2012). These findings suggest that teamwork modulates UT responses by improving providers’ uncertainty tolerance (Kenen et al., 2011; Page & Mander, 2014; Persson et al., 2011).

Healthcare systems may be well placed to help adjust the moderator of role ambiguity. Herein, it was found that a lack of role clarity resulted in lower uncertainty tolerance. This is consistent with many reports that lack of clarity on healthcare roles creates uncertainty (Acker, 2003; Chang & Hancock, 2003; Fackler et al., 2009; Page & Mander, 2014). To address this, healthcare systems could create onboarding and ongoing support focused on improved role clarity, particularly as roles change and evolve over time. Examples include interprofessional orientations and education workshops.

### Uncertainty tolerance as modifiable construct

The findings from this systematic review suggest that UT, as a whole, may be a modifiable construct with a trait and a state aspect. We identified intrinsic and extrinsic factors which modulate providers’ responses to healthcare uncertainty, suggesting that UT responses are, at least in part, contextually determined. We did identify some evidence of a trait focused portion of the model in Domain 1. Herein, personal characteristics appear unchanged by the state or context in which the healthcare uncertainty is stimulated. Our findings in Domain 2 and Domain 3 mainly identified moderators in the Stimulus Characteristics and Situational Characteristics of the IMUT, both of which are contextual, meaning they are modifiable, as opposed to non-modifiable moderators. This further supports our postulation that there is both a trait and state aspect of the UT construct. Based on our findings across the three domains, where in part, moderators in Domain 1 are state-based, while moderators in Domains 2 and 3 are all state-based, it suggests that the trait aspect of UT may sit as a personal characteristic “moderator” while the surrounding characteristics in the environment are the state aspect of UT. As such, UT perceptions and responses may, on the whole, be state-specific, but the traits of the individual may inherently modulate the response towards one response over another.

### Limitations and future research

While this work cannot capture the full range of moderators, the intent was to provide a starting point for characterizing moderators in the IMUT. The moderator characterizations are limited to the data available in published peer-reviewed reports and theses and is likely not an exhaustive representation of all potential moderators. Future studies would be well placed to focus on prospective data collection exploring moderators in the healthcare environment. A potential limitation of this review is the exclusive focus and evaluation of qualitative studies, and concomitant exclusion of quantitative studies. While quantitative studies could offer additional evidence and insight into moderators, UT survey items often conflate moderators with responses making it difficult to tease out the impact of the stimulus versus moderator with item analysis alone (Strout et al., 2018). Furthermore, by identifying moderators through healthcare provider’s descriptions and experiences of UT, it is likely that we may not have uncovered the cultural and societal influences that may affect how a healthcare provider responds to UT.

It is also important to acknowledge that the IMUT suggests that moderators can influence uncertainty tolerance at two stages: 1) between the ambiguous stimulus and the perception of the stimulus being uncertain, and 2) between the perception of the stimulus being uncertain and the response (cognitive, emotional and behavioral). As the IMUT did not discuss either moderator influencing stages in great detail, we chose to focus on the second stage of the model, looking at how we could characterize moderators and understand the relationship they had on responses to uncertainty. Further studies are required to refine and understand the first stage of where the moderators can act on between the ambiguous stimuli and perception of the stimuli being uncertain.

Uncertainty as a modifiable construct is an interesting concept as this suggests that we have the potential to increase individuals’ tolerance to uncertainty through moderators. The literature, as well as our findings show that there are many negative outcomes associated with those who are less tolerant of uncertainty which supports a narrative that being tolerant is more desirable than being less tolerant. However, Reis-Dennis et al., (2021) suggest that uncertainty tolerance is not so black and white, and that there are nuances regarding the advantages associated with being tolerant to uncertainty, and those who are highly tolerant are likely to also experience disadvantages (such as complacency which eventuates into poor clinical practice). Reis-Dennis et al., (2021) suggest that there are certain personality traits (moderators) that could be cultivated such as courage, diligence, and curiosity to manage the dangers around excessive tolerance or intolerance to uncertainty. As such, in this context, it suggests that further research is required to provide us with additional insights as to what an ‘optimal’ level of uncertainty tolerance is, and when it would be appropriate to cultivate uncertainty tolerance as being tolerant of uncertainty may not necessarily equate to positive responses.

As the interpretations of the data are limited to the studies that are currently available, while we can identify links between moderators and responses, we cannot comment when there is lack of available data. The IMUT proposes a role for moderators at both the perception and response phases of UT. Because no evaluated studies explored real-time perceptions and/or responses to uncertainty (e.g., talk-aloud protocols), and given that the IMUT proposes moderators impacts on both of these phases, we were often unable to explicitly determine whether moderators were acting solely on individuals’ responses or were acting on both their perception and responses. In investigating the links between moderators and responses, many of the articles in this review exclusively report negative appraisals of UT, and thus there may be a bias of negative effects of identified moderators. For instance, articles included in this review only reported when healthcare providers did not disclose uncertainty to patients. While this may represent a reality that only negative appraisals are observed with certain moderators, a more likely explanation is that the positive responses are under-reported and are not generally described and thus further purposeful investigation into potential positive influences of moderators on providers’ UT may be required. We acknowledge that our review focuses on the experiences of UT from a providers’ perspective and not a patient’s perspective and would recommend future studies to explore our research question from a patient perspective as healthcare uncertainty is prevalent from both perspectives. Further research efforts will be well invested in defining a clearer boundary between the uncertainty stimulus and moderating ‘stimulus characteristics’ as well as exploring which parts of the model are “state/trait-based.”

Due to the potential dual influence that moderators have in the integrative model; 1) influencing the individuals’ perception of the uncertainty stimulus; and 2) an individuals’ response to the stimulus, in our included articles, it was sometimes challenging to differentiate when a moderator was affecting the participants’ perception of the stimulus or whether it was influencing the participants’ response *to* the stimulus. As such, we recommend future research explore the nuances around the influences of a moderator to help clarify which stage (perception or response) that the moderator is acting on.

Further research exploring the range of the behavioral response of “referral” also appears warranted. Patient referral appears to be a behavioral outcome across all three moderator domains. Previous studies suggests that presentation complexity tends to result in patient referrals (Persson et al., 2011; Morgan et al., 2007). Referral outcomes may present as an appropriate solution for further patient evaluation or may be an inappropriate step by a doctor to help mitigate their own intolerance of healthcare uncertainty, and the difference between these two determine the referral behavioral response. Furthermore, our review raises questions around whether the ease of referrals could, in and of itself, be a moderator of healthcare providers’ UT. Further research, thus, would be well placed to look more closely at UT through the lens of decision-making and appropriateness of providers’ referrals to shed light on this potentially powerful proxy measure of doctors’ UT. In doing so, follow-up research may be able to inform strategies for evaluating the efficacy of interventional measures targeting improvements to providers’ uncertainty tolerance.

## Conclusion

In this study, we have investigated the moderators of uncertainty tolerance in healthcare. Our research question was: ‘*What moderators of healthcare professionals’ tolerance of uncertainty within the clinical context are currently described, and how are they defined?’*, with our research aims focusing on; 1) further characterization of moderators of healthcare UT (Hillen et al., 2017); and 2) identify moderators impacts on the Cognitive, Emotional and Behavioral responses to uncertainty in healthcare professionals. We explored our research question by conducting a systematic review of qualitative literature which reported primary accounts of healthcare professionals’ descriptions of uncertainty. Our findings further contribute to the debate of whether UT is a trait-based or state-based construct. In particular, our findings suggest that UT as a whole has both trait-based and state-based components, as the moderators identified in our three domains illustrate that individuals’ responses to uncertainty vary based on their individual characteristics, situational characteristics and stimulus characteristics. Some of these moderators (such as confidence and cultural competence) have been shown to improve with practice or experience which suggests that they are modifiable, while other moderators such as presentation complexity is a situational characteristic that is not modifiable. Ultimately, this work also contributes more broadly towards facilitating our understanding of the complex nature of the UT construct, and in doing so, adds to UT theory development.

## Funding source

None.

### Supplementary Information

Below is the link to the electronic supplementary material.

Supplementary file1 (DOCX 20 KB)
