# Supplementary material for: Moderators uncertainty tolerance (UT) in healthcare: a systematic review
Source: Adv Health Sci Educ Theory Pract. 2023 Apr 25;28(5):1409–40. doi: 10.1007/s10459-023-10215-0 (PMC10700225; doi:10.1007/s10459-023-10215-0)
Supplement: Supplementary file 1 — Supplementary file1 (DOCX 20 KB) [file 10459_2023_10215_MOESM1_ESM.docx]

*Supplementary Table 1.* *Quality appraisals of the 17 included articles using the CASP tool.*

|  | Q1 Clear research aims | Q2 Appropriate qualitative methodology | Q3  Appropriate research design | Q4  Appropriate recruitment strategy | Q5  Collected data addresses research issue | Q6  Researcher and participant relationship | Q7  Ethical issues considered | Q8  Rigorous data analysis | Q9  Clear statement of findings | Q10  Contributions of study to field |
| --- | --- | --- | --- | --- | --- | --- | --- | --- | --- | --- |
| Andre (2016) | Yes | Yes | Can't tell | Yes | Yes | No | yes | Yes | Yes | Yes |
| Borg (2010) | Yes | Yes | Yes | Yes | Yes | Can't tell | Yes | Can't tell | Yes | Yes |
| Bouchard (2016) | Yes | Yes | Yes | Yes | Yes | Can't tell | Yes | Can't tell | Can't tell | Yes |
| Causey (2011) | Yes | Yes | Yes | Yes | Yes | Can't tell | Yes | Yes | Yes | Yes |
| Dogra (2007) | Yes | Yes | Yes | Yes | Yes | Yes | Yes | Yes | Yes | Yes |
| Fackler (2009) | Yes | Yes | Yes | Yes | Yes | Can't tell | Yes | Can't tell | Yes | Yes |
| Gowda (2018) | Can't tell | Yes | Yes | Yes | Yes | Can't tell | Yes | Yes | Yes | Yes |
| Ilgen, (2020) | Yes | Yes | Yes | Yes | Yes | Can't tell | Can't tell | Yes | Yes | Yes |
| Kenen (2011) | Yes | Yes | Yes | Yes | Yes | Can't tell | Yes | Yes | Yes | Yes |
| Knight (2016) | Yes | Yes | Can't tell | Can't tell | Can't tell | Can't tell | Yes | Yes | Yes | Yes |
| Morgan (2007) | Yes | Yes | Yes | Yes | Yes | Yes | Yes | Can't tell | Yes | Yes |
| Nurse-Clarke (2021) | Yes | Yes | Yes | Yes | Yes | Can't tell | Can't tell | Yes | Yes | Yes |
| Nevalainen (2009) | Yes | Yes | Yes | Yes | Yes | Yes | Yes | Yes | Yes | Yes |
| Page (2014) | Yes | Yes | Yes | Yes | Yes | Can't tell | Yes | Yes | Yes | Yes |
| Persson (2011) | Yes | Yes | Yes | Yes | Yes | Yes | Yes | Yes | Yes | Yes |
| Roeske (2013) | Yes | Yes | Yes | Yes | Yes | Yes | Yes | Yes | Yes | Yes |
| van Iersel (2019) | Yes | Yes | Yes | Yes | Yes | Can't tell | Yes | Can't tell | Yes | Yes |
